# Além do Colesterol e dos Triglicerídeos na Clínica Lipídica: A Desafiadora Tarefa de Identificar a Deficiência de Lipase Ácida Lisossomal

**DOI:** 10.36660/abc.20250359

**Published:** 2025-08-06

**Authors:** Marjorie H. Mizuta, Raul D. Santos

**Affiliations:** 1 Hospital das Clínicas da Faculdade de Medicina da Universidade de São Paulo Instituto do Coração São Paulo SP Brasil Instituto do Coração do Hospital das Clínicas da Faculdade de Medicina da Universidade de São Paulo, São Paulo, SP – Brasil; 2 Hospital Israelita Albert Einstein São Paulo SP Brasil Hospital Israelita Albert Einstein, São Paulo, SP – Brasil

**Keywords:** Colesterol, Triglicerídeos, Doença de Wollman

A deficiência de lipase ácida lisossomal (LAL-D) é uma doença genética rara, autossômica recessiva. Variantes patogênicas bialélicas no gene *LIPA* resultam em uma deficiência da enzima lipase ácida lisossomal (LAL), crucial para a hidrólise de ésteres de colesterol (EC) pela via endocítica. Esse defeito enzimático, consequentemente, leva ao acúmulo lisossomal de EC e triglicerídeos. O fenótipo da LAL-D varia significativamente dependendo da variação genética específica. Especificamente, variantes alélicas nulas, caracterizadas pela ausência de função enzimática residual, manifestam-se como doença de Wolman, enquanto variantes com alguma atividade enzimática residual da LAL resultam em doença de acúmulo de ésteres de colesterol (CESD). Notavelmente, identificar indivíduos com LAL-D representa um desafio significativo, dada a heterogeneidade fenotípica da doença, que pode potencialmente se sobrepor a vários diagnósticos diferenciais.^1^

A doença de Wolman geralmente se manifesta na infância. A apresentação clínica inclui vômitos, esteatorreia e distensão abdominal nos primeiros dias de vida.^2^ Hepatoesplenomegalia pode ocorrer devido ao depósito de lipídios no fígado e no baço, e a esteatose hepática pode levar à insuficiência hepática. O acúmulo de EC e triglicerídeos no trato gastrointestinal leva ao espessamento da parede intestinal, que por sua vez causa má absorção e subsequente desnutrição. A calcificação das glândulas suprarrenais é uma característica clássica da doença e pode levar à insuficiência cortical adrenal. Indivíduos com doença de Wolman raramente sobrevivem além do primeiro ano de vida, principalmente devido à desnutrição grave, insuficiência hepática e insuficiência cortical adrenal. Embora a terapia de reposição enzimática seja considerada, a expectativa de vida permanece limitada devido à gravidade da doença.^1^ O transplante de células-tronco hematopoiéticas representa outra opção terapêutica; no entanto, mais estudos são necessários devido aos achados conflitantes relatados na literatura.^3 , 4^

A CESD representa uma forma mais branda de LAL-D com um espectro clínico variável que abrange da infância à idade adulta. O fenótipo inclui dislipidemia, elevação das enzimas hepáticas, diarreia e perda de peso; calcificação adrenal também pode ocorrer. Na LAL-D, o acúmulo lisossomal de EC e triglicerídeos leva a uma redução no colesterol livre citosólico, causando regulação positiva compensatória da atividade da hidroximetilglutaril-CoA (HMG-CoA), o que resulta em níveis elevados de colesterol total e LDL. Também pode inibir a acil-CoA: colesterol aciltransferase, levando a uma diminuição nos níveis de colesterol HDL. Xantelasmas e hipertrigliceridemia também podem estar presentes.^1^ A CESD também pode levar à hepatoesplenomegalia, resultando potencialmente em insuficiência hepática.^1 , 5^ A deposição de lipídios gastrointestinais pode causar diarreia e perda de peso.^6^ Pode ocorrer aumento da glândula adrenal com calcificações puntiformes, tipicamente em indivíduos com um fenótipo grave.^7^ Apesar da heterogeneidade fenotípica, a CESD geralmente tem um prognóstico mais favorável do que as formas graves de LAL-D.^1 , 5^

A identificação de portadores de LAL-D continua sendo um desafio. Embora a doença de Wolman e a CESD difiram clinicamente, testes genéticos, que apresentam limitações e podem não estar disponíveis, são frequentemente necessários para um diagnóstico definitivo. Além disso, os sintomas variados da CESD frequentemente levam a diagnósticos equivocados como outros distúrbios do metabolismo lipídico, incluindo hipercolesterolemia familiar, deficiência de esfingomielinase ácida (doença de Niemann-Pick tipos A e B) ou doença de Gaucher.^1 , 8^

Na edição atual da ABC, Brasil et al. avaliaram retrospectivamente registros de 2.018 adultos e crianças de uma clínica de lipídios usando um algoritmo de triagem.^9^ Os autores selecionaram 21 pacientes com alto risco para LAL-D para teste de atividade de LAL. No entanto, apenas oito pacientes foram submetidos ao teste, com todos os resultados sendo normais. Uma criança apresentou atividade de LAL indetectável post-mortem, e a triagem em cascata familiar subsequente identificou portadores de LAL-D dentro da família. Este estudo destaca os desafios do diagnóstico de LAL-D, mesmo em grupos de alto risco. Dada sua raridade e a necessidade de exclusão, os autores usaram cuidadosamente outros testes para descartar condições semelhantes. Como limitações do estudo, o algoritmo empregado não havia sido validado para precisão ou capacidade discriminatória, e as enzimas hepáticas foram obtidas sob tratamento com estatina, o que pode ter superestimado a seleção desses indivíduos.

O diagnóstico de LAL-D é estabelecido pela identificação de variantes patogênicas bialélicas ou provavelmente patogênicas no gene *LIPA* ou pela atividade deficiente da enzima LAL em leucócitos do sangue periférico, fibroblastos ou manchas de sangue seco. Apesar de sua etiologia genética, a LAL-D nem sempre pode ser diagnosticada por testes genéticos devido às limitações, incluindo o tipo de teste genético e a presença de variantes potencialmente desconhecidas. Brasil et al. ilustraram isso em seu estudo, pois nenhuma variante patogênica foi identificada no gene *LIPA* .^9^ A baixa atividade da enzima LAL confirma a LAL-D, mas não distingue a doença de Wolman da CESD, pois os sintomas podem variar mesmo com níveis enzimáticos semelhantes. Além disso, a mesma variante genética pode levar a níveis variáveis de atividade enzimática.^1^ Semelhante à triagem em cascata molecular usada em famílias com distúrbios monogênicos, Brasil et al. utilizaram de forma inovadora uma abordagem em cascata reversa, medindo a atividade enzimática, permitindo um diagnóstico preciso dentro da família estudada.^9^ Considerando os desafios diagnósticos, estudos adicionais são necessários para corroborar a identificação de indivíduos com LAL-D. Enquanto isso, medidas estratégicas, incluindo testes em cascata reversa (enzimáticos ou genéticos) iniciados em probandos mais jovens, a inclusão de *LIPA* em painéis abrangentes de dislipidemia e a avaliação de consanguinidade na família, podem nos ajudar a orientar em direção a um diagnóstico.
