# Supplementary material for: Além do Colesterol e dos Triglicerídeos na Clínica Lipídica: A Desafiadora Tarefa de Identificar a Deficiência de Lipase Ácida Lisossomal
Source: Arq Bras Cardiol. 2025 Aug 6;122(7):e20250359. [Article in Portuguese] doi: 10.36660/abc.20250359 (PMC12695102; doi:10.36660/abc.20250359)
Supplement: Translation

# Translation: Beyond Cholesterol and Triglycerides in the Lipid Clinic: The Challenging Task of Identifying Lysosomal Acid Lipase Deficiency

**DOI:** 10.36660/abc.20250359i

**Authors:** Marjorie H. Mizuta, Raul D. Santos

**Affiliations:** 1 São Paulo SP Brazil Instituto do Coração do Hospital das Clínicas da Faculdade de Medicina da Universidade de São Paulo, São Paulo, SP – Brazil; 2 São Paulo SP Brazil Hospital Israelita Albert Einstein, São Paulo, SP – Brazil

**Keywords:** Cholesterol, Triglycerides, Wolman's disease

Lysosomal acid lipase deficiency (LAL-D) is a rare, autosomal recessive genetic disorder. Biallelic pathogenic variants within the *LIPA* gene result in a deficiency of the lysosomal acid lipase (LAL) enzyme, which is crucial for the hydrolysis of cholesteryl esters (CE) via the endocytic pathway. This enzymatic defect consequently leads to the lysosomal accumulation of CE and triglycerides. The phenotype of LAL-D varies significantly depending on the specific genetic variation. Specifically, null allelic variants, characterized by the absence of residual enzymatic function, manifest as Wolman's disease, while variants with some residual LAL enzymatic activity result in cholesterol ester storage disease (CESD). Notably, identifying individuals with LAL-D poses a significant challenge, given the phenotypic heterogeneity of the disease, which can potentially overlap with various differential diagnoses.^1^

Wolman's disease usually manifests in infancy. The clinical presentation includes vomiting, steatorrhea, and abdominal distension within the first few days of life.^2^Hepatosplenomegaly may occur due to lipid deposition in the liver and spleen, and hepatic steatosis can lead to liver failure. The accumulation of CE and triglycerides in the gastrointestinal tract leads to intestinal wall thickening, which in turn causes malabsorption and subsequent malnutrition. Calcification of the adrenal glands is a classic feature of the disease and can lead to adrenal cortical insufficiency. Individuals with Wolman's disease rarely survive beyond their first year of life, primarily due to severe malnutrition, liver failure, and adrenal cortical insufficiency. Although enzyme replacement therapy is considered, life expectancy remains limited due to the disease's severity.^1^Hematopoietic stem cell transplantation represents another therapeutic option; however, further studies are necessary due to conflicting findings reported in the literature.^3 , 4^

CESD represents a milder form of LAL-D with a variable clinical spectrum spanning from childhood to adulthood. The phenotype includes dyslipidemia, elevated liver enzymes, diarrhea, and weight loss; adrenal calcification may also occur. In LAL-D, the lysosomal accumulation of CE and triglycerides leads to a reduction in cytosolic free cholesterol, causing compensatory upregulation of hydroxymethylglutaryl-CoA (HMG-CoA) activity, which results in elevated total and LDL cholesterol levels. It may also inhibit acyl-CoA: cholesterol acyltransferase, leading to a decrease in HDL cholesterol levels. Xanthelasmas and hypertriglyceridemia may also be present.^1^CESD may also lead to hepatosplenomegaly, potentially resulting in liver failure.^1 , 5^Gastrointestinal lipid deposition can cause diarrhea and weight loss.^6^Adrenal gland enlargement with punctate calcifications may occur, typically in individuals with a severe phenotype.^7^Despite phenotypic heterogeneity, CESD generally has a more favorable prognosis than severe forms of LAL-D.^1 , 5^

Identifying LAL-D carriers remains challenging. While Wolman's disease and CESD differ clinically, genetic testing, which has limitations and may be unavailable, is often needed for a definitive diagnosis. Furthermore, CESD's varied symptoms often lead to misdiagnosis as other lipid metabolism disorders, including familial hypercholesterolemia, acid sphingomyelinase deficiency (Niemann-Pick disease types A and B), or Gaucher's disease.^1 , 8^

In the current issue of ABC, Brasil et al. retrospectively assessed records from 2,018 adults and children from a lipid clinic using a screening algorithm.^9^The authors selected 21 patients at high risk for LAL-D for LAL activity testing. However, only eight patients underwent the test, with all results being normal. One child had undetectable LAL activity post-mortem, and subsequent family cascade screening identified LAL-D carriers within the family. This study highlights the challenges of diagnosing LAL-D, even in high-risk groups. Given its rarity and the need for exclusion, the authors carefully used other tests to rule out similar conditions. As study limitations, the employed algorithm had not been validated for accuracy or discriminatory ability, and liver enzymes were obtained under statin treatment, which could have overestimated the selection of these individuals.

LAL-D diagnosis is established by identifying biallelic pathogenic or likely pathogenic variants in the *LIPA* gene or by deficient LAL enzyme activity in peripheral blood leukocytes, fibroblasts, or dried blood spots. Despite its genetic etiology, LAL-D cannot always be diagnosed by genetic testing due to limitations, including the type of genetic testing and the presence of potentially unknown variants. Brasil et al. illustrated this in their study, as no pathogenic variants were identified in the *LIPA* gene.^9^Low LAL enzyme activity confirms LAL-D but does not distinguish Wolman's from CESD because symptoms can vary even with similar enzyme levels. Additionally, the same genetic variant can lead to varying enzyme activity levels.^1^Similar to molecular cascade screening used in families with monogenic disorders, Brasil et al. innovatively utilized a reverse cascade approach by measuring enzymatic activity, enabling an accurate diagnosis within the studied family.^9^Given the diagnostic challenges, further studies are warranted to corroborate the identification of individuals with LAL-D. In the interim, strategic measures, including reverse cascade testing (enzyme or genetic) initiated in younger probands, the inclusion of *LIPA* in comprehensive dyslipidemia panels, and assessing consanguinity within the family, may help guide us toward a diagnosis.
